# Association between Inflammatory Cytokine Levels and Thrombocytopenia during* Plasmodium falciparum* and* P. vivax* Infections in South-Western Coastal Region of India

**DOI:** 10.1155/2019/4296523

**Published:** 2019-04-11

**Authors:** Kishore Punnath, Kiran K. Dayanand, Valleesha N. Chandrashekar, Rajeshwara N. Achur, Srinivas B. Kakkilaya, Susanta K. Ghosh, Suchetha N. Kumari, D. Channe Gowda

**Affiliations:** ^1^Department of Biochemistry, K. S. Hegde Medical Academy, NITTE (Deemed to be University), Mangaluru, India; ^2^Department of Biochemistry, Kuvempu University, Shankaraghatta, Shivamogga District, Karnataka, India; ^3^Light House Polyclinic, Light House Hill Road, Mangaluru, Karnataka, India; ^4^Department of Molecular Parasitology, ICMR-National Institute of Malaria Research, Poojanahalli, Bangalore, India; ^5^Department of Biochemistry and Molecular Biology, The Pennsylvania State University College of Medicine, 500 University Drive, Hershey, PA, USA

## Abstract

**Background:**

Thrombocytopenia is a most commonly observed complication during malaria infections. Inflammatory cytokines such as IL-1, IL-6, and IL-10 have been documented in malaria induced thrombocytopaenia. This study was aimed to understand the possible relationship between inflammatory cytokines across varying degrees of thrombocytopenia during* P. vivax*,* P. falciparum,* and mixed infections.

**Methods:**

A hospital-based cross sectional study was conducted at District Wenlock Hospital in Mangaluru, a city situated along the south-western coastal region of Arabian Sea in India. In this study, blood samples from 627 malaria patients were analyzed for infected parasite species, clinical conditions, platelet levels, and key cytokines that are produced in response to infection; samples from 176 uninfected healthy individuals were used as controls.

**Results:**

The results of our study showed a high prevalence of malarial thrombocytopenia (platelets <150 ×10^3^/*μ*l) in this endemic settings. About 62.7% patients had mild-to-moderate levels of thrombocytopenia and 16% patients had severe thrombocytopenia (platelets <50 × 10^3^/*μ*l). Upon comparison of cytokines across varying degrees of thrombocytopenia, irrespective of infecting species, the levels of TNF-*α* and IL-10 were significantly higher during thrombocytopenia, whereas IL-6 levels were considerably lower in severe thrombocytopenia patients suffering from* P. vivax *or* P. falciparum* infections. The severe clinical complications observed in patients with malarial thrombocytopenia included severe anemia (17.5%), acute renal failure (12.7%), jaundice (27.0%), metabolic acidosis (36.5%), spontaneous bleeding (3.2%), hypoglycemia (25.4%), hyperparasitemia (4.8%), acute respiratory distress syndrome (1.6%), pulmonary edema (19.0%), and cerebral malaria (1.6%) in various combinations.

**Conclusion:**

Overall, the results of our study suggest that inflammatory cytokines influence the transformation of mild forms of thrombocytopenia into severe forms during malarial infections. Further studies are needed to understand the association of inflammatory cytokine responses with severe malaria complications and thrombocytopenia.

## 1. Introduction

Protozoan parasites of the genus* Plasmodium* cause malaria, a devastating disease prevalent across tropical regions around the world. In the year 2016, more than 216 million clinical cases and 445,000 deaths were reported across 91 countries worldwide [1]. Nearly half of the world's population is at risk of malaria infections. In Southeast Asia alone, >1.4 million clinical cases and >550 deaths occur every year due to malaria. In India, during 2016, ~1.09 million clinical cases and 331 deaths were reported [1].

Mangaluru is the administrative headquarters of Dakshina Kannada district of Karnataka state in southern India. This coastal city is surrounded by Netravati and Gurupura rivers and is located between the waters of Arabian Sea and hills of Western Ghats. Since 1930, Mangaluru city has been fighting malaria and is still considered to be endemic in this region [2, 3]. In 2017, among the 11312 malarial cases reported in the Karnataka state, Mangaluru alone contributed to 8075 (71.4%) cases. Two major species of* Plasmodium*, namely,* P. vivax,* Pv, (6452, 79.9%) and* P. falciparum*, Pf, (1623, 20.1%) infections are prevalent in the city and its surrounding regions. The malarial infections are common throughout the year in this region with its peak transmission during the rainy season, between the months of June to September [3]. The factors that contribute to high prevalence of malaria in this region are warm and humid tropical climate, high rainfalls, rapid economic development, and vast urbanization.

It is well known that, proinflammatory cytokines play an important role in clearance of malaria parasites. However, a well-timed and balanced pro- (Th1 type) and anti-inflammatory (Th2 type) cytokine release are critical for favorable outcome of the disease [4]. The asexual blood stage infection manifests in wide range of clinical symptoms, including periodic fever and chills, headache, malaise, cough, abdominal pain, and diarrhea. Studies have suggested that imbalanced inflammatory responses particularly during high parasitic burden can aggravate the malaria symptoms and results in serious pathological complications such as severe anemia (SA), acute respiratory distress syndrome (ARDS), acute renal failure (ARF), and cerebral malaria (CM) [5, 6]. The proinflammatory cytokines such as tumor necrosis factor alpha (TNF-*α*), interferon gamma (IFN-*γ*), and interleukin-12 (IL-12) aid in inhibiting parasite growth and stimulate monocyte phagocytosis to enhance clearance of parasitized erythrocytes [7]. Inflammatory cytokines such as IL-17 and IL-22 contribute to inflammation by recruitment of neutrophils and induction of secretion of several proinflammatory cytokines. The timely regulation by anti-inflammatory cytokines such as IL-10, IL-4, and IL-13 to control the production and possible cytopathic effects of proinflammatory cytokines, plays a major role in limiting the progression of the uncomplicated malaria into its severe forms [8, 9].

Apart from their classical function to maintain homeostasis during injury, platelets are also known to play important roles in immune response to infection and the pathogenesis of malaria by promoting the sequestration of infected red blood cells (iRBCs) in the vasculature of the brain and other organs [10–12]. Recent evidence suggests that platelets also have the ability to kill* Plasmodium *parasites [13]. Thrombocytopenia is a common hematological alteration during malaria infections which is characterized by decreased platelet levels (<150 × 10^3^/*μ*l). Although thrombocytopenia is commonly observed during* Plasmodium* infections, the mechanisms leading to this complication are not well defined. Thrombocytopenia seems to occur primarily by peripheral destruction, bone marrow alterations, excessive removal of platelets by splenic pooling, platelet consumption by the process of disseminated intravascular coagulopathy (DIC), antibody-mediated platelet destruction, pseudo thrombocytopenia due to clumping of* P. falciparum *infected erythrocytes, and oxidative stress [14].

Although it has long been perceived that only Pf infections cause severe thrombocytopenia, recent studies suggest that this clinical condition is also common in* P. vivax* (Pv) and* P. knowlesi* (Pk) infections [15–20]. Thrombocytopenia is also considered as a sensitive diagnostic marker for malaria [21]. Several studies have reported on thrombocytopenia associated pathologies during malaria infections in various endemic regions [16, 17]. However, only a limited number of studies have analyzed the levels of inflammatory cytokines during malarial thrombocytopenia. We recently reported on the pathological conditions and biochemical parameters of a randomly recruited group of inpatients and outpatients with Pf, Pv, and mixed (Pf and Pv) infections who were treated at the Government Wenlock Hospital in Mangaluru city. In this study, the samples were analyzed for the prevalence and assessment of degree of thrombocytopenia, as well as for cytokines such as TNF-*α*, IL-6, and IL-10. The results of these analyses are presented here.

## 2. Materials and Methods

A prospective hospital-based study was carried out at the District Wenlock Hospital in Mangaluru during November 2013 to October 2015. This Government hospital provides free medical treatment for patients from nearby locations of 7 districts from the states of Karnataka and Kerala. A total of 803 individuals aged between 15 and 65 years were randomly recruited into the study. The study protocol was approved by the ethical committee of Kuvempu University, Shivamogga, Karnataka state, the central ethics committee of NITTE University, Mangaluru, and the Institutional Review Board of Pennsylvania State University College of Medicine, Hershey, PA, USA. All the participants were orally explained about the study and were recruited upon obtaining informed consent from study participants or their relatives. All the infected patients had the classical malarial symptoms such as intermittent fever, chills, and rigors and were treated at the outpatient and inpatient departments of District Wenlock Hospital in Mangaluru. The individuals attending blood bank for blood donations and testing negative for malaria were enrolled as healthy controls (HC). Exclusion criteria included pregnant women, use of any antipyretics prior to diagnosis and individuals testing positive for dengue, typhoid, human immunodeficiency virus (HIV), and hepatitis B and C infections.

The malarial infections were confirmed by careful microscopic examination of Giemsa stained peripheral blood smears. Two thick and thin blood slides were prepared from each study participant, stained with 4% Giemsa stain and observed under the microscope for the presence of* Plasmodium*, and identification of the parasite species type. The parasite densities were determined as parasites/*μ*l of blood (number of parasites counted/number of white blood cells, WBCs, counted × total number of WBCs per *μ*l of blood) or (number of parasites counted/ number of red blood cells-RBCs counted × total number of RBCs per *μ*l of blood). Percentage of parasitemia was determined as number of parasites per *μ*l of blood/number of RBCs per *μ*l of blood× 100. No PCR analyses were carried out for malarial diagnosis.

Before giving any antimalarial medications, about 2-3 ml of venous blood was drawn aseptically into sterile heparin coated vacutainers for plasma preparation and into clot activator tubes for serum preparation and kept at 4°C. After centrifugation, serum and plasma samples were prepared, labeled and stored at −70°C until further use. The infected patients were treated as per the National Vector Borne Disease Control Program [NVBDCP] recommendations [22]. The* P. vivax-*infected patients were treated with a combination of chloroquine for 3 days and primaquine for 14 days and the individuals with* P. falciparum *infections were treated with artemisinin-based combination therapy (artesunate plus sulphadoxine-pyrimethamine) and primaquine as a single dose. The patients who had severe malaria complications and required supportive treatment were admitted to the hospital and treated by the attending physician.

The platelet count, mean platelet volume (MPV), plateletcrit (PCT), and platelet distribution width (PDW) were determined by using automated hematology analyzer (Mind Ray-Biomedical, Shenzhen, China). The analysis of cytokines, TNF-*α*, IL-6, and IL-10, in plasma was performed in duplicate by sandwich ELISA using kits from R&D Biotech, USA, as per manufacturer's instructions. Recombinant human cytokines were used to obtain standard curves ranging from 9.38 to 2000 pg/ml.

### 2.1. Classification of Study Participants

The study participants were grouped into (i) HC, individuals testing negative for presence of* Plasmodium* parasites, (ii) uncomplicated malaria (UM), the patients who had low grade fever, headache, or chills and positive for the presence of* Plasmodium* parasite by peripheral blood smear (these patients were treated on an outpatient basis) and (iii) severe malaria (SM), patients requiring hospital admission and supportive care due to severe malarial complications, as per WHO guidelines, such as severe anemia (Hb<5 g/dl), acute renal failure (serum creatinine >3mg/dl), jaundice (serum bilirubin >3mg/dl), metabolic acidosis (plasma bicarbonate <15mmol/l), spontaneous bleeding, hypoglycemia (plasma glucose <40 mg/dl), hyperparasitemia (>5% parasitemia), ARDS, pulmonary edema, and CM [23]. Thrombocytopenia is defined as a decrease in platelet counts of <150 × 10^3^/*μ*l. Based on the levels of platelets, the thrombocytopenic patients were grouped into (i) non-thrombocytopenia (NT, >150 × 10^3^/*μ*l), (ii) mild thrombocytopenia (MT, 100-150 × 10^3^/*μ*l), (iii) moderate thrombocytopenia (MDT, 50-100 × 10^3^/*μ*l), and (iv) severe thrombocytopenia (ST, <50 × 10^3^/*μ*l).

### 2.2. Statistical Analysis

The statistical analysis was performed using Graphpad Prism version-6 (Graphpad Prism software Inc., San Diego, CA, USA) and R version 3.4.2 (https://www.r-project.org/). Quantitative variables are presented as mean ± standard deviation and mean ± interquartile range. Summary statistics were determined for baseline demographics and quantitative variables. The comparison of nonparametric data between various groups was performed by Kruskal-Wallis Test and significance between two groups was determined by Mann-Whitney U Test with 95% confidence interval with adjustments for multiple testing. Spearman rank correlation was used to determine correlations between two continuous variables. The equality of proportions or percentages was determined by a three-sample test without continuity correction. If found significant, a two-sample binomial proportion test was used to obtain the significant highest proportion.* P *values less than 0.05 were considered to be significant.

## 3. Results

### 3.1. Demographics of Study Participants

A total of eight hundred and three (n=803) individuals comprising of 627 (78.1%) malarial infected patients and 176 (21.9%) as HC were enrolled in the study at Government Wenlock Hospital in Mangaluru city. Briefly, the mean age of the study participants (including HC) was 30.3 years (age range, 16 to 65 years). The majority of infected individuals were males (402, 64.1%) and the numbers of females were 225 (35.9%). Among the 627 infected patients, 554 (88.4%) patients had MM and were treated on an outpatient basis and 73 (11.6%) patients required admission due to SM complications. Among the various infecting species, Pv was the most prevalent species of infection (n=384, 61.3%) followed by Pf (n=172, 27.4%) and mixed (n=71, 11.3%) infections (Table 1).

### 3.2. Platelets and Inflammatory Cytokines Profile of the Study Participants

The levels of RBC, hemoglobin, platelets, platelet indices, and plasma levels of inflammatory cytokines were compared between (i) HC and infecting groups and (ii) between various infecting groups. The mean parasitemia in patients with* P. falciparum* (0.8±1.15%) was higher than mixed (0.6±0.75%) and* P. vivax* (0.3±0.49%) groups, indicating a higher parasitic burden during Pf infections (Table 2). A significant decrease in RBC counts was observed across all infecting species in comparison with HC (p<0.0001); within the various infecting species, Pf infection resulted in significantly decreased RBC counts. A significant negative correlation was observed between RBC levels and parasitic burden during Pf (r=-0.0218, p=0.0076) and Pv (r=-0.1322, p=0.0104) infections. Hemoglobin levels were significantly decreased across various infecting species in comparison to HC (p<0.0001), and within different infecting species, the hemoglobin levels were significantly decreased among Pf patients (Table 2). A significant negative correlation between increased parasitemia and decreased hemoglobin levels was observed across all patients regardless of infected parasite species; Pv (r=-0.3145, p<0.00001), Pf (r=-0.2863, p=0.0003), and mixed infections of Pv and Pf (r=-0.2671, p=0.0359). Compared to HC, the platelet levels were significantly decreased in all infecting groups (p<0.0001), especially during Pf infections (Table 2). In all the three infected groups, platelet levels were decreased as parasitemia increased during Pv (r=-0.2140, p=0.0003), Pf (r=-0.1929, p=0.0169), and mixed (Pv and Pf) infections (r=-0.1170, p=0.0053). The plasma levels of inflammatory cytokines were significantly increased upon malarial infections. The levels of TNF-*α*, IL-6, and IL-10 were significantly increased in comparison with HC (p<0.0001) (Table 2). There was no statistically significant influence of age and gender on the levels of RBC, hemoglobin, platelets, platelet indices, and plasma levels of inflammatory cytokines analyzed (p>0.05).

### 3.3. Classification of Thrombocytopenia Intensity among Study Participants

Among the 176 HC included in this study, a majority 168 (95.5%) were non-thrombocytopenic (NT) whereas 6 (3.4%) had mild thrombocytopenia (MT) and 2 (1.1%) had moderate thrombocytopenia (MDT). Of the total 627 infected patients, 493 (78.6%) patients had varying levels of thrombocytopenia; 134 (21.4%) were NT, 188 (30%) had MT, 205 (32.7%) had MDT, and 100 (15.9%) had severe thrombocytopenia (ST) (Table 3).

### 3.4. Comparison of Cytokine Levels across Varying Degrees of Thrombocytopenia

**Figure 1:**
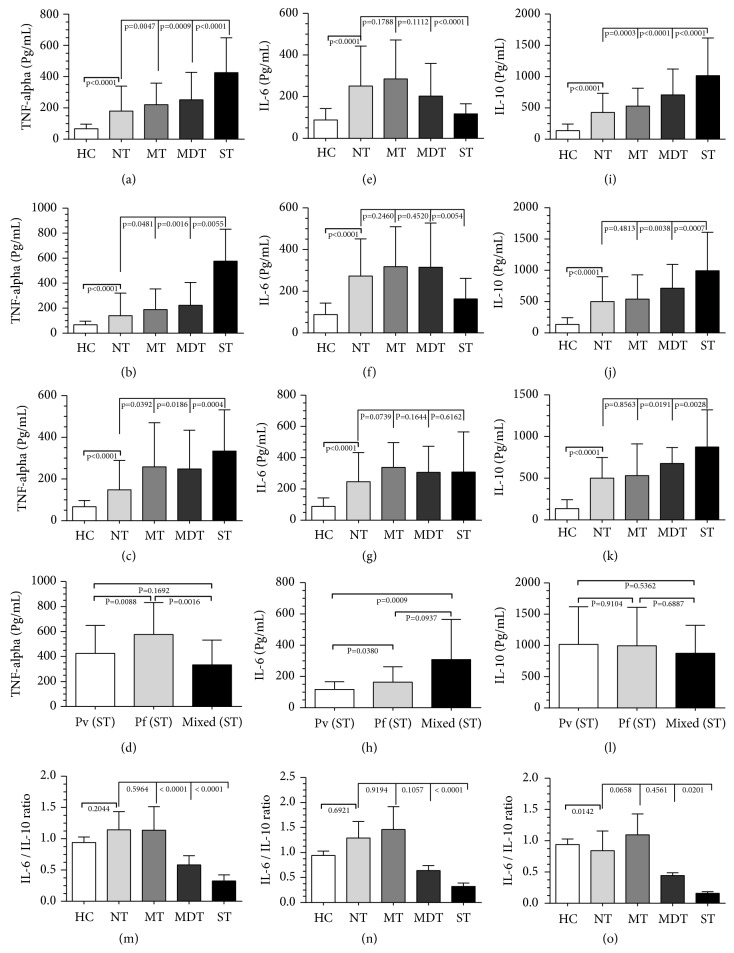
*Inflammatory cytokine levels during varying degrees of malarial thrombocytopenia*. Plasma levels of TNF-*α*, IL-6, and IL-10 during* P. vivax* (a, e, i),* P. falciparum* (b, f, j), and mixed (c, g, k), and the cytokine levels of TNF-*α* (d), IL-6 (h), and IL-10 (l) in ST patients. Ratios of IL-6 and IL-10 across varying degrees of thrombocytopenia during* P. vivax* (m),* P. falciparum* (n), and mixed (o).Data shown as median (inter quartile range 25% and 75%) and were analyzed by one-way nonparametric Kruskal-Wallis test for multiple comparisons and Mann Whitney U test for comparison between two groups;* P* values <0.05 were considered to be significant.

The mean platelet and plasma cytokine levels across varying degrees of thrombocytopenia during Pv, Pf, and mixed infections were compared and analyzed (Table 4, Figure 1). In comparison with NT groups, the TNF-*α* levels were found to be gradually increased with increasing intensity of thrombocytopenia during Pv, Pf, and mixed infections (p<0.05). Between the ST groups of various infecting species, TNF-*α* levels were found to be significantly increased in patients with Pf infections (*P*-value = 0.0032). The IL-6 levels, in comparison with NT groups, were found to be decreased in ST patients during Pv and Pf infections. Upon comparison within ST groups across infecting species, the IL-6 levels were significantly lower in Pv patients (*P*-value = 0.0023). Irrespective of infecting species, the IL-6/IL-10 ratio also showed a significant decrease with an increase in thrombocytopenic intensity, especially during severe thrombocytopenia.

In comparison with NT groups, the IL-10 levels were found to be significantly increased across varying intensity of thrombocytopenia during Pv, Pf, and mixed infections. However, within the ST groups across various infecting species, the IL-10 levels did not show any significant change (Table 4,* P*-value = 0.8379).

### 3.5. Clinical Manifestations in Patients with Malarial Thrombocytopenia

Among the 176 HC, 168 (95.5%) were NT and 8 (4.5%) had mild-to-moderate level of thrombocytopenia. Of 627 malaria patients, 493 (78.6%) patients had thrombocytopenia. Among these thrombocytopenic patients, 63 (12.7%) patients required hospital admissions due to severe malarial complications such as severe anemia (17.5%), acute renal failure (12.7%), jaundice (27.0%), metabolic acidosis (36.5%), spontaneous bleeding (3.2%), hypoglycemia (25.4%), hyperparasitemia (4.8%), acute respiratory distress syndrome (1.6%), pulmonary edema (19.0%), and cerebral malaria (1.6%) in various combinations (Table 5). All SM patients were treated successfully and there were no deaths.

## 4. Discussion

This study has analyzed the levels of platelets, inflammatory cytokines, and severe malarial complications in patients with thrombocytopenia during malaria infections. A significant decrease in platelet levels, especially during mixed infections and an inverse relationship between parasitemia and platelet counts across various infecting species, was observed as reported earlier [24, 25]. The mean MPV levels increased as platelet counts fell across all infecting groups which could be due to early release of giant platelets (megakaryocytes) from the bone marrow to compensate the reduced platelet levels, thus preserving primary homeostasis and avoiding severe bleeding [26–28].

In this endemic setting, overall only 21.4% patients were non-thrombocytopenic, whereas 30% of patients had mild thrombocytopenia, 32.7% had moderate thrombocytopenia, and 15.9% had severe thrombocytopenia. Regardless of the infected parasite species, thrombocytopenia was frequently detected: Pv (80.5%), Pf (79.1%), and mixed infections (67.6%). These findings are comparable to previously published observations from Pv endemic settings [25]. Though the thrombocytopenia per se cannot differentiate between the infecting parasite species, the high prevalence of thrombocytopenia could be a potential marker for malarial infections [16, 21, 24].

The cytokines released during inflammatory response in malaria could contribute to the severity of thrombocytopenia. To understand the influence of key cytokines in thrombocytopenia, the plasma levels of TNF-*α*, IL-6, and IL-10 were compared and analyzed in patients with varying degrees of thrombocytopenia during Pv, Pf, and mixed infections. TNF-*α* is an important inflammatory cytokine known to influence a wide variety of responses. Elevated TNF-*α* levels induce thrombocytopenia resulting in platelet trapping and consumption that occurs in inflamed blood vessels [29]. Various* in vivo* studies reveal that the mice injected with TNF-*α* results in decreased platelet levels, suggesting that TNF-*α* is associated with platelet consumption [30, 31]. In our study, as reported earlier, the TNF-*α* levels were found to be significantly increased with increasing severity of thrombocytopenia across all infecting species [29, 32]. Thus, elevated TNF-*α* levels may imply a similar role in patients with severe thrombocytopenia.

IL-6 promotes megakaryocytopoiesis* in vitro* and raise platelet counts* in vivo* [32–37]. It is well known that IL-6 upregulates the thrombopoietin (TPO) levels leading to proliferation and maturation of megakaryocytes* in vivo*, which in turn results in increased platelet levels [38–42]. Our observations are in contrast with earlier studies which reported increased IL-6 levels [43]. However, in studies with contrasting results, the IL-6 levels were analyzed among the overall thrombocytopenic patients. In our study, upon analyzing cytokine levels among patients with varying intensity of thrombocytopenia, the IL-6 levels did not change significantly during mild and moderate thrombocytopenia but were found to be decreased during ST in patients with* P. vivax* and* P. falciparum* infections [44]. These lower IL-6 levels during ST could have been inadequate for TPO upregulation and in turn the reason behind the decreased platelet levels [37].

IL-10 is an anti-inflammatory cytokine, primarily produced by macrophages are known to inhibit Th1 responses [45]. Administration of human recombinant IL-10 in healthy volunteers leads to decreased platelet levels. This decrease is attributed due to decreased proinflammatory cytokine production from monocytes and macrophages, which in turn leads to decreased hematopoietic progenitor cells such as megakaryocyte colony-forming units (CFU-MKs), in turn affecting the platelet production [46]. In this study, similar to earlier reports, patients with severe thrombocytopenia were also found to have increased IL-10 levels across various infecting species, implying a similar role of IL-10 in decreased platelet levels [43, 44, 47, 48].

Despite not being a criterion for severe malaria recommended by WHO, several life-threatening clinical conditions, including disseminated intravascular coagulation (DIC), platelet associated IgG increase, immune thrombocytopenia purpura, acute renal failure, pulmonary edema, splenomegaly, and cerebral malaria have been reported in patients with severe thrombocytopenia [49–51]. In the present study, among the thrombocytopenic patients admitted, severe malarial complications such as severe anemia, acute renal failure, jaundice, metabolic acidosis, spontaneous bleeding, hypoglycemia, hyperparasitemia, acute respiratory distress syndrome, pulmonary edema, and cerebral malaria in various combinations were observed. Clinical cases of bleeding or biochemical evidence of DIC, though associated with severe thrombocytopenia are not commonly observed. In our study, only two ST patients with mixed infections experienced bleeding manifestations and 6 patients were given platelet transfusions.

## 5. Conclusions

In consistent with the previous findings, our study suggests that Pv infections can also result in a similar degree of severe thrombocytopenia as observed in Pf and Pk infections. We also found that patients with thrombocytopenia, irrespective of infecting species, experienced severe malarial complications such as severe anemia, acute renal failure, jaundice, metabolic acidosis, spontaneous bleeding, hypoglycemia, hyperparasitemia, acute respiratory distress syndrome, pulmonary edema, and cerebral malaria. The results also suggest a possible role of cytokines such as TNF-*α*, IL-6, and IL-10 in decreased or disturbed platelet production, resulting in malarial thrombocytopenia. In conclusion, as these lines of studies are scarce in India, studies in other regions are warranted to support the present findings of thrombocytopenia during malaria. Further, studies are needed to understand the role of inflammatory cytokines and its association with severe malarial complications during thrombocytopenia in malaria especially during* P. vivax *infections.

## 6. Limitations

Although the study is extensive, there are some limitations such as i) only adults were recruited into the study excluding children; ii) the malarial diagnosis was performed only by Giemsa stained peripheral blood smear and no polymerase chain reaction (PCR) analysis was performed.

## Figures and Tables

**Table 1:** Characteristics of study participants across various infecting species.

	Healthy controls	*P. vivax*	*P. falciparum*	Mixed	Overall Infected	p value^a^
Number of study participants, n (%)	176	384(61.3)	172 (27.4)	71 (11.3)	627 (100)	<0.0001
Uncomplicated malaria (UM)	0	351 (63.4)	149 (26.9)	54 (9.7)	554 (88.4)	<0.0001
Severe malaria (SM)	0	33 (45.2)	23 (31.5)	17 (23.3)	73 (11.6)	0.6853
Gender, n (%)						
Males	116	246 (61.2)	111(27.6)	45 (11.2)	402(64.1)	0.9866
Females	60	138 (61.3)	61 (27.1)	26(11.6)	225 (35.9)	0.9866
Age (in years, mean, range)	30.1 (16-58)	30.5 (16-65)	32.7 (16-65)	31.7 (16-65)	30.3 (16-65)	>0.05

Data represented as number of study participants (percentages) ^a^p values upon comparison between three infecting groups from multinomial proportion test.

**Table 2:** Changes in hematological parameters and inflammatory cytokines during malarial infection.

Parameter	Normal range	Healthy controls	*P. vivax*	*P. falciparum*	Mixed	P value^a^ (between groups)		
						*Pv *Vs *Pf*	*Pv *Vs Mixed	*Pf Vs Mixed*
Parasitemia (%)			0.3 ± 0.49	0.8 ± 1.15	0.6±0.75	< 0.001	< 0.0001	0.5957
RBC	4.0-6.0 X 10^3^/*μ*l	5.0 ± 0.73	4.7 ±0.92	3.7 ± 0.91	4.8 ±1.98	0.0011	0.5994	0.0485
Hemoglobin	Males: 14-18; Females: 12-16 g/dl	12.5 ± 1.26	11.5 ± 2.86	10.1 ± 2.93	10.7 ±3.22	0.0003	0.0699	0.0383
Platelets	100 - 400 x10^3^/*μ*l	210 ± 57.2	108.2 ± 55.3	92 ± 45.1	101 ±75.3	0.0095	0.7869	0.036
MPV	6.5-12.0 fL	9.4 ± 1.03	10.3 ± 1.63	10.2 ±1.36	10.5 ±1.56	0.7358	0.0216	0.0218
PDW	9.0 - 17.0 %	14.7 ± 3.79	14.9 ± 0.89	14.7 ±1.23	15.0 ±1.19	0.6861	0.479	0.3722
PCT	0.108 - 0.282 %	0.2±0.06	0.1±0.05	0.1±0.05	0.1±0.06	0.9063	0.1112	0.1761
TNF-*α* (pg/ml)		67.0±29.5	251.8±101.2	259.9±144.0	236.7±190.2	0.1402	0.2536	0.8992
IL-6 (pg/ml)		88.1±54.8	227.4±63.9	280.2±44.4	292.9±94.2	0.0013	0.0038	0.7219
IL-10 (pg/ml)		136.0±66.2	634.9±83.1	671.2±68.6	688.4±43.2	0.6299	0.1061	0.3904

Data represented as mean ± SD; comparison of two groups by Mann-Whitney U Test; ^a^P value > 0.05 was considered to be significant. RBC: Red blood cells, MPV: Mean platelet volume, PDW: Platelet distribution width, PCT: plateletcrit, TNF-*α*: Tumor necrosis factor- alpha, IL-6: interleukin-6, IL-10: interleukin-10.

**Table 3:** Stratification of study participants according to the varying intensity of thrombocytopenia.

Thrombocytopenia intensity	Healthy controls	*P. vivax*	*P. falciparum*	Mixed	Overall Infected	p value^a^
Non-thrombocytopenia –NT (platelet levels >150 x10^3^/*μ*l)	168 (95.5)	75 (19.5)	36 (20.9)	23 (32.4)	134(21.4)	0.065
Mild thrombocytopenia – MT (platelet levels 100-150x10^3^/*μ*l)	6 (3.4)	127 (33.1)	48 (27.9)	13 (18.3)	188 (30)	0.055
Moderate thrombocytopenia –MDT (platelet level 50-100x10^3^/*μ*l)	2 (1.1)	129 (33.6)	57 (33.1)	19 (26.8)	205 (32.7)	0.512
Severe thrombocytopenia –ST (platelet levels <50x10^3^/*μ*l)	0(0)	53 (13.8)	31 (18.1)	16 (22.5)	100 (15.9)	0.279

Data represented as number of study participants (percentages) and was compared between three infecting groups by using values from multinomial proportion test. ^a^P value> 0.05 was considered to be significant.

**Table 4:** Cytokines during varying thrombocytopenia levels during patients with *P. vivax*, *P. falciparum,* and mixed infections.

Cytokines	*P. vivax*			
	NT	MT	MDT	ST
n (%)	75 (19.5%)	127 (33.1%)	129 (33.6%)	53 (13.8%)
Platelets x10^3^/*μ*l	183 (162-203)	124 (112-134)	79 (67-90)	30(22-40)
TNF-*α* (pg/ml)	131.7 (70.6-220.7)	208.0 (108.1-312.0)	213.0 (106.0-362.8)	431.9 (232.4-607.6)
IL-6 (pg/ml)	208.3 (109.0-366.6)	264.0 (154.3-402.4)	173.7 (111.8-249.8)	111.2 (79.9-158.5)
IL-10 (pg/ml)	373.9 (256.1-493.4)	502.1 (352.7-671.4)	641.8 (441.6-892.3)	892.8 (545.5-1556.0)

Cytokines	*P. falciparum*			

n (%)	36 (20.9%)	48 (27.9%)	57 (33.1%)	31 (18.0%)
Platelets x 10^3^/*μ*l	166 (159-191)	127 (116-135)	80 (68-92)	29 (13-38)
TNF-*α* (pg/ml)	82.0 (51.0-127.2)	151.8 (56.2-249.5)	193 (78.2-312.5)	602.2 (359.4-761.6)
IL-6 (pg/ml)	247.0 (136.0-370.7)	324.1 (160.2-424.4)	284 (151.8-456.1)	140.4 (116.6-165.6)
IL-10 (pg/ml)	429.7 (169.7-701.5)	486.4 (267.0-751.0)	679.8 (372.2-978.9)	874.9 (348.5-1585.5)

Cytokines	Mixed			

n (%)	23 (32.4%)	13 (18.3%)	19 (26.8%)	16 (22.5%)
Platelets x 10^3^/*μ*l	162 (154-182)	118 (118-136)	76 (56-84)	29 (20-36)
TNF-*α* (pg/ml)	83.8 (67.0-160.4)	154.2 (135.3-371.6)	189.1 (107.6-339.0)	284.9 (185.7-473.3)
IL-6 (pg/ml)	237.0 (93.1-308.5)	325.1 (227.3-403.9)	270.5 (153.5-417.7)	241.6 (115.6-380.7)
IL-10 (pg/ml)	506.4 (298.7-729.6)	531.3 (263.7-639.4)	722.1 (509.6-813.5)	883.6 (591.5-1064.1)

Data shown as median (inter quartile range 25% and 75%) and were analyzed by one-way nonparametric Kruskal-Wallis test for multiple comparisons and Mann Whitney U test for comparison between two groups.

**Table 5:** Severe malarial complications in severe thrombocytopenia patients across various infecting species.

Clinical condition	Total (n=63)	*P. vivax* (n=28)	*P. falciparum* (n=20)	Mixed (n=15)	p value^a^
Severe anemia	11 (17.5)	4 (14.3)	4 (20)	3 (20)	0.499
Acute renal failure	8 (12.7)	4 (14.3)	2 (10)	2 (13.3)	0.063
Jaundice	17 (27.0)	6 (21.4)	5 (25)	6 (40)	0.063
Metabolic acidosis	23 (36.5)	12 (42.9)	8 (40)	3 (20)	0.827
Spontaneous bleeding	2 (3.2)	0 (0)	0 (0)	2 (13.3)	NA
Hypoglycemia	16 (25.4)	7 (25)	6 (30)	3 (20)	0.134
Hyperparasitemia	3 (4.8)	0 (0)	3 (15)	0 (0)	NA
Acute respiratory distress syndrome (ARDS)	1 (1.6)	1 (3.6)	0 (0)	0 (0)	NA
Pulmonary edema	12 (19)	5 (17.9)	4 (20)	3 (20)	0.498
Cerebral malaria (CM)	1 (1.6)	1 (3.6)	0 (0)	0 (0)	NA

Data represented as number of patents (percentage) and was compared between three infecting groups by using multinomial proportion test. ^a^P < 0.05 was considered to be significant.

## Data Availability

The data used to support the findings of this study are available from the corresponding author upon request.

## Abbreviations

Pv: * P. vivax*
Pf: * P. falciparum*
Pk: * P. knowlesi*
NVBDCP: National Vector Borne Disease Control Program
UM: Uncomplicated malaria
SM: Severe malaria
WBCs: White blood cells
RBCs: Red blood cells
Hb: Hemoglobin
HCT: Hematocrit
MPV: Mean platelet volume
PDW: Platelet distribution width
CM: Cerebral malaria
ARDS: Acute respiratory distress syndrome
T: Thrombocytopenia
NT: Nonthrombocytopenia
ST: Severe thrombocytopenia.
